# Dermatology

**Published:** 1904-12

**Authors:** W. Kenneth Wills


### DERMATOLOGY.

In the section for Dermatology at the Oxford meetings a most interesting comparison was drawn by Dr. J. H. Sequeira[1] between the old and new methods of treatment of **lupus vulgaris** and certain other skin diseases. So much has been written upon this subject, and by so many different authors, that to obtain the scientific judgment of so experienced and sound an observer must prove a great gain to all who are following with interest the recent work in these directions. The comparison is made by putting to each method the questions: (1) Is it curative? (2) What will be the amount of disfigurement?

Excision in early cases, where practicable, is undoubtedly the most radical, but is not always curative (48 per cent., Lang); but great disfigurement must ensue upon the ablation of the large tracts necessary for the relief of most of the cases which appear for treatment. It is practicable mainly for the trunk and extremities.

Scraping in the main has proved most unsatisfactory. The nodules are removed, but not the infected skin surrounding them. The lymphatic spaces are torn open in a ruthless way, and the curettes are plunged from one nodule to another, and it is not surprising that spreading of the disease should follow operation. Scraping should be limited to the removal of exuberant granulations, and possibly the destruction of nodules embedded in scar tissue. It should always be followed by the application of caustics or cautery. The scars left are very unsightly.

It is hard to limit the deep action necessary for the destruction of nodules with caustics or cautery; but their use is helpful in fungating granulations in the nose and about the mouth. Sequeira has found pyrogallic acid and permanganate of potash useful in thick warty masses as well as in fungating granulations. Their application is painful, and not deep enough for deep nodules.

Scarification produces a beautiful scar, but is tedious and superficial. The Finsen treatment is undoubtedly the best method of treatment for lupus of the skin of the face, though there are certain limitations. There is a direct sterilisation of the tissues, with a minimum of disfigurement. At the margin sterilisation takes place without scarring. The Finsen and the newer Finsen-Reyn lamps are the only instruments which penetrate sufficiently to reach the deeper nodules. With the Lortet lamp the sittings must last for an hour as a rule to ensure penetration, and then only superficial lupus can be cured. Concentration of light is important.

The idea of sensitising the tissue to light is a good one. Sequeira has tried some cases with 0.2 per cent. erythrosin

[1] *Brit. M. J.*, 1904, ii. 982.

(Dreyer) The reaction is intense, but there is severe pain, and further observations are necessary.

Mucous membranes require other methods of treatment. Ulcerous surfaces need preliminary treatment, and thick warty masses must be removed by pyrogallic or other caustic. Some very extensive cases have proved intractable. Results since May, 1900:—

COMPLETED CASES.

| | |
|---|---|
| Patients known to be well to date— | |
| Discharged in 1900–01 ... ... ... ... | 49 |
| ,, ,, 1902 ... ... ... ... ... | 60 |
| ,, ,, 1903 ... ... ... ... ... | 69 |
| ,, ,, 1904 (half year) ... ... ... | 38 |
| | 216 |
| Patients discharged, but no recent information ... | 8 |
| Died of phthisis, 2; general tuberculosis, 1 ... ... | 3 |
| Incurable after prolonged treatment by light ... ... | 11 |
| | 238 |

INCOMPLETE CASES.

| | |
|---|---|
| Treatment suspended—under observation ... ... | 43 |
| Patients left for treatment elsewhere ... ... ... | 22 |
| ,, ,, for other causes (domestic, &c.) ... ... | 23 |
| Treatment incomplete (in a large number skin free, mucous membranes lesions requiring treatment) | 213 |
| | 539 |

The X-rays are of immense value in the treatment of lupus vulgaris. Large areas can be treated; ulcerated parts heal rapidly, but nodules are left isolated, which require other methods of treatment. The scar produced may be excellent; but on the other hand it may be covered with telangiectases, which are disfiguring. The recent report of cases of epithelioma produced upon the hands of X-ray workers makes it possible that X-ray scars may become malignant under prolonged treatment. Sequeira clears up ulcerating forms with X-rays, and finishes with light. There are frequent relapses. The results of the light alone are greatly superior to that of X-rays where it is applicable.

High-frequency treatment is useful for ulcerated surfaces, but not for nodules. He sums up his choice of measures as follows:—

For moderate cases of lupus of the skin of the face Finsen treatment, preceded by X-rays if the surface is ulcerated, preceded by pyrogallic acid or permanganate of potash if warty or fungating.

For lupus of mucous membranes X-rays; if fungating, actual cautery followed by X-rays.

For dry areas on the limbs and trunk, excision and, if necessary and possible, grafting; if ulcerated, X-rays.

For wide dissemination probably the best results are to be expected from tuberculin injections, as recently demonstrated by Dr. Wright.

**Rodent Ulcer.**—Small ulcers may be excised or treated with radium. Ulcerations treated with X-rays heal; raised edges may be scraped away, and then treated with X-rays. Radium is occasionally successful where X-rays fail. Epithelioma may develop on rodent; but this may be due to natural causes, and not necessarily to the X-rays.

With regard to epithelioma his experience has been discouraging. Superficial epitheliomata have been removed, but glandular masses are not affected. Radium has removed small warty epitheliomata which develop upon old cases of the red variety of lupus.

**Mycosis Fungoides.**—Further cases of this disease have been found amenable to treatment by means of the X-rays.

Jamieson[1] records a case of the erythro-dermic stage which was relieved entirely by this method. An attack of influenza brought out lesions again in a part which had not been rayed, and these were relieved in their turn by the same agency. All trace of the disease, with its glandular enlargement, had disappeared, leaving only slightly rough skin on abdomen and thighs. The microscopic findings from portions removed showed the typical granulomata, and others the complete removal thereof.

Carrier[2] has also been successful with a case, the X-rays removing the tumours and causing a cessation of the spread. The treatment lasted daily for five minutes at a time from August 17th to November 10th, and the result was, there were no induration, scaling, erythema, tumours, or pruritus.

**Alopecia Areata.**—L. Jacquet[3] has found it impossible to inoculate the disease in six persons, one of whom had previously suffered from it and might therefore have proved susceptible. The inoculations were made by collecting on a moistened tampon of cotton-wool the supposed contagious products obtained by scraping and vigorous friction of the areas and their borders, which was then rubbed into the parts of the scalp preferred by the disease.

Thirty of the writer's temporo-parietal hair follicles were catheterised with an electrolysis needle charged with the products of the scraping of another case. One hundred inoculations in all were conducted, but not a hair was lost, the results being absolutely *nil*.

Jacquet has also drawn attention to the frequency with which disturbances in dentition are associated with bald areas,

[1] *Brit. J. Dermat.*, 1904, xvi. 125.
[2] *J. Cutan. Dis.*, 1904, ii. 73; abstract in *Brit. M. J.*, 1904, i. Epitome, p. 59.
[3] *Presse méd.*, 1903, ii. 857; abstract in *Brit. J. Dermat.*, 1904, xvi. 195.

and Whitfield[1] has lately found that some cases of alopecia areata proved intractable to treatment until the eyesight had been corrected and eyestrain removed.

Kromayer[2] has treated six cases of extensive and severe alopecia areata which had resisted such treatment as mercury spirit, chrysarobin ointment, or eugallol, with the rays from an iron-electrode lamp. Exposing for four minutes at 4 c.m. distance the whole area was treated for fifteen sittings. The reactions were good, and in one case were kept up by these means for fourteen days, and then left for a while and repeated later. This was from February 25th to May 12th. By June the whole of the hair had returned luxuriantly. Of the other five cases, one was cured after one cycle, one after three cycles, and one after six, and the last after one. In one case recurrence occurred in six months, but the renewal of the treatment was successful.

**Megalerythema Epidemicum, or Erythema Infectiosum.**—Under this name Plachte[3] describes a disease characterised by rounded patches of erythema raised and bright red in colour, with edges not sharply defined, without pain or itching, and showing an infectious tendency among children. The erythema is not persistent, but tends to fade in the centre of the initial patches, and to spread to other parts of the body. There is very little if any desquamation. In the cases he brings forward three children in one family suffered, aged 7 and 3 (twins). The mucous membranes were not involved and there was no pyrexia, but there were slight pains in the limbs. The general health appeared to be affected in no other way. The rash appeared first on the face and later on the body, tending here to become confluent. The disease resembles erythema multiforme, but can be distinguished from it by the following points of diagnosis:—

(1) E-multiforme affects the whole body simultaneously, whereas megalerythema affects certain positions in a definite order; (2) E-multiforme does not occur as an epidemic; (3) the spots of megalerythema are circular and do not present the variety of E-multiforme; (4) the duration of E-multiforme is 2–6 weeks, that of megalerythema nine days.

Other observers have reported similar cases, the types of which correspond to Plachte's case, though there are variations such as itching, sore throat, and initial rise of temperature.

**Purpura.**—Henri Grenet[4] considers that the nervous system plays a very decided part in every case of purpura. He records six cases of exanthematous purpura which showed nervous reactions. Three patients presented simultaneously albumin and leucocytosis in the cerebro-spinal fluid drawn by means of

[1] *Lancet*, 1904, i. 651.
[2] *Deutsche med. Wchnschr.*, 1904, xxx. 1127; abstract in *Brit. M. J.*, 1904 ii. Epitome, p. 39.
[3] *Berl. klin. Wchnschr.*, 1904, xli. 223. [4] *Gaz. d. Hôp.*, 1904, lxxvii. 868.

lumbar puncture. In one case the purpura alternated with an attack of zona. In another herpes of head and eyes rapidly ensued on the purpura dying away; and in still another, a child of a hæmophilic mother, there was lymphocytosis and a thick cloud of albumin in the cerebro-spinal fluid. On improvement the albumin persisted, but was explained four days later by an attack of herpes on the face. On the subsidence of this the albumin was reduced to a faint trace, and the lymphocytes nearly disappeared. The writer considers that this contravenes M. Leredde's belief that the purpura is solely due to the action of toxins circulating in the blood upon the vessel walls.

**Treatment.**—In excessive sweating of the feet Weiss[1] recommends the use of potassium permanganate in the following way:—Having cleansed the feet with borax, water, soap, and cotton pledget with benzine, they are immersed in vessels containing enough 1 per cent. permanganate to reach to the ankles. After fifteen minutes the feet are lightly dried and dusted with pot. permang., alum, talc, zinc oxide, and lapis calaminaris as a powder, the toes being kept separate with cotton-wool. This is repeated each night for fourteen days, the strength of the permanganate being gradually increased to ℥i to the quart. Weiss urges that this treatment is, unlike chromic acid, devoid of danger; is painless, it can be used without fear when there are fissures, as it heals them without the need of silver nitrate; it can be used in every stage of the disease; relapses are less common than with other treatments, rarely occurring before the third month; the use of the dusting powder will prolong the period before recurrence; the desquamation is not attended by tenderness, and walking is not unpleasant as with chromic acid. The treatment is not unpleasant to undergo except in so far as the excessive staining of the skin and nails is concerned. Corns are shed easily while the treatment is being used.

Dreuw,[2] in commenting upon the use of yeast in skin treatment, states that it contains albumin, fats, carbohydrates, water, salts with nuclein, zymose, endotrypsin and katalase.

Nuclein, by its action in producing leucocytosis after absorption through the gastro-intestinal tract, and its power of neutralising and destroying certain bacterial toxins, is of therapeutic importance. It is of use in abscess, acne, suppurating conditions of the skin, erysipelas, folliculitis, impetigo, and urticaria.

Yeast may be applied externally or internally. To facilitate the external application he has made a soap containing the active principles of yeast and one which is overfatted. It can be applied by (1) lathering and washing off, (2) lathering and drying in air and then rubbing with a flannel or woollen

[1] *J. Am. M. Ass.*, 1904, xliii. 369.

[2] *Deutsche med. Wchnschr*, 1904, xxx. 991; abstract in *Brit. M. J.*, 1904, ii. Epitome, p. 28.

cloth, (3) allowing suds to dry under a water-tight dressing. The intensity is in direct proportion to the method adopted. He has had the soap medicated with ac. salicyl, sulphur, ichthyol, &c. He claims good results with acne, folliculitis, small furuncles, and chronic dry eczema. In furunculi and folliculitis he recommends the salicylic and sulphur yeast soap.

W. KENNETH WILLS.

### Reviews of Books.

